# Bet More – But Only with Me: Role of Celebrity Endorsers in Sports Betting Advertising

**DOI:** 10.1007/s10899-025-10399-7

**Published:** 2025-06-17

**Authors:** Wenjia Han, Vaidyanathan Viswanathan Saunak

**Affiliations:** 1 Department of Hospitality and Tourism Management, Doermer School of Business, Purdue University Fort Wayne, Fort Wayne, IN 46805 USA; 2 Department of Management and Marketing, Doermer School of Business, Purdue University Fort Wayne, Fort Wayne, IN 46805 USA

**Keywords:** Responsible gambling, Brand intention, Celebrity endorser, Image congruence, Sports betting, Social norms

## Abstract

Since the federal ban on sports betting was lifted in 2018, the industry has grown rapidly in the United States, raising concerns about the risk of problem gambling. Building on existing research that suggests the limited effectiveness of traditional responsible gambling campaigns, the current study examines whether the use of celebrity endorsers in advertising can enhance responsible gambling intentions. Using a scenario-based experiment with 383 U.S. sports bettors, regression analyses reveal that an endorser whose image aligns with the target audience increases consumer awareness of responsible gambling messages, thereby enhancing intentions to gamble responsibly. In addition, an image-congruent endorser boosts consumers’ betting intentions and word-of-mouth advocacy for the sportsbook. Although image congruence in advertising is often operationalized through racial similarity, the findings suggest that same-race endorsers are effective in enhancing image congruence only when sports betting is perceived as socially acceptable. This study is the first to examine the effect of endorser-consumer image congruence on consumer intentions toward both sports betting brands and responsible gambling. The findings provide important insights to sportsbooks aiming to balance branding and social responsibility in marketing campaigns.

## Introduction

As an integral component of the entertainment industry, gambling is often discussed for its societal costs, including increased gambling addiction and criminal activity (Lind, 2022; Nikkinen & Marionneau, 2020). The expansion of internet-based services has further intensified concerns due to the broader accessibility and anonymity of online gambling (Rosenbaum & Russell-Bennett, 2020). Despite these concerns, the U.S. Supreme Court’s decision in 2018 to overturn the federal ban on sports wagering has transformed the industry landscape. As of 2024, 38 states and Washington D.C. have legalized sports wagering, either online or in-person (Petrella, 2024). This legalization has fueled rapid industry growth, and major sportsbook operators have been competing for market shares with aggressive marketing and advertising campaigns. As of 2023, more than half of American adults live in an environment where sports betting is legal (American Gaming Association, 2023a, b). While sportsbook revenue has surged more than 30-fold from 2018 ($0.4 billion) to 2024 ($14.2 billion) and provided $2.9 billion in state and local tax in 2024 alone (Ramsey, 2025), social concerns about problem gambling have grown. The National Council on Problem Gambling reported a 30% rise in gambling addiction risks from 2018 to 2021, yet less than half of individuals know where to seek help (Huble, 2023). To address social concerns, the American Gaming Association advocates that sportsbooks follow the Responsible Marketing Code for Sports Wagering, which requires advertisements to include a clearly visible responsible gaming message and a toll-free helpline number (American Gaming Association, 2023b).

Responsible gambling (RG) messages in advertisements are added to minimize the related harm while promoting gambling activities. However, research suggests that the RG messages in commercial ads may be ineffective in enhancing the awareness of responsible gambling and driving behavior change (Lole et al., 2019). A recent study identified viewers’ psychological reactance and inattentiveness as the primary reasons for the limited effectiveness of RG messages (McCullock et al., 2024). The similarity-attraction effect in psychology suggests that people tend to be attracted to those perceived to be similar to themselves (Hampton et al., 2019). Therefore, this study examined whether target consumers would exhibit stronger responsible gambling intentions when a celebrity endorser who resembles them appears in the advertisement. Grounded in self-congruity theory, the current study proposes that an endorser whose image is congruent with the target consumer may serve as an exemplar, thereby encouraging responsible gambling behavior. In addition, this effect is further amplified when paired with a responsible gambling (RG) message, as an image congruent endorser reduces viewers’ psychological reactance and inattentiveness.

While the effective advocacy of RG is essential, the primary objective of commercial advertisement remains to increase brand awareness and promote brand usage. Therefore, a commercial sports betting advertisement should not only convey responsible gambling messages but also positively affect consumers’ behavioral intentions toward the brand. Accordingly, this study also examined whether an image-congruent endorser enhances consumers’ intentions to learn more about, place bets with, and speak favorably about the sportsbook being advertised. While self-congruity theory contends that consumers tend to develop positive attitudes toward products with a brand-user image congruent with themselves (Kim, 2015), social identity theory suggests that customers’ decision-making depends on whether significant others in their social circle encourage the intended behavior (Champniss et al., 2015). Even though the sports betting business boosts the economy, about one-third of U.S. adults perceive its widespread legalization as negative for society and sports (Gramlich, 2022). Terlutter et al. (2022) found that consumers respond negatively to advertisements featuring same-race endorsers engaging in socially disapproved behavior. The phenomenon implies the need to examine the moderating effect of social norms on the relationship between endorsers who resemble the consumer in appearance and the consumer’s self-identity.

Responsible gambling information is critically important in the design of ad campaigns for sportsbooks. The current research examines the effect of image-congruent endorsers in enhancing ad viewers’ intention to gamble responsibly. In addition, it examines the effect of image-congruent endorsers in enhancing consumer behavioral intentions through ads. It also investigates the role of social norms in forming the perceived image congruence between the endorser and consumers. The findings of this research bridge the gaps in the gambling literature by examining the effect of endorser image congruence on promoting social responsibility messages and enhancing consumer behavioral intentions towards a gambling product. This study provides important insights to marketers and policymakers on planning responsible gambling campaigns.

## Literature Review

### Celebrity Endorsement and Consumer Behavioral Intentions

Sports betting advertisements often resonate with the audience by appealing to the sports culture (McMullan & Miller, 2008). The positive attributes of sports games are emphasized in sports betting advertisements, such as competition, skill, talent, sports spirit, etc. (Lopez-Gonzalez et al., 2017). Celebrities, especially athletes, are frequently used as endorsers because of their inspiring achievements (Belch et al., 2013). Research applying eye-tracking techniques found that, compared to unknown characters, the appearance of celebrities in advertising expedites consumers’ decision-making (D’Ambrogio et al., 2023). In the gambling-related literature, research on the symbolic construction of sports betting suggests that athletes serve as a medium through which the skill-based attributes of sports are transferred to gambling products, thereby reducing the perceived risk associated with sports betting (Lopez-Gonzalez et al., 2021). Scholars also found that celebrity endorsement is effective only when implemented properly, which includes selecting celebrities who align with the product and brand image, as well as ensuring that the celebrity’s public image is maintained well throughout the endorsement period (Yang, 2018). Consumer purchase behavior can be negatively influenced if either of the two conditions is not met (Yang, 2018).

Research focusing on the hospitality industry summarized three perspectives to understand the effectiveness of celebrity endorsement. The source attractiveness perspective argues that celebrity is a credit source that enhances consumers’ trust in the brand and confidence in product quality. The match-up hypothesis contends that a good fit between the product and the celebrity drives the effectiveness of the endorsement (Xu et al., 2018). The meaning transfer model suggests that consumers define their self-identity through consumption, where the association that the ad builds between the celebrity and the product is subconsciously transferred to the establishment of consumers’ self-image (Xu et al., 2018). Accordingly, the congruence between the celebrity and the consumer’s self-concept further motivates consumers to purchase the endorsed products to act like the celebrity (Choi & Rifon, 2012).

In typical sports wagering advertisements, a bettor is depicted as a tech-savvy and professional male, which is an image that appeals to the target audience of sports betting (Hing et al., 2016). Supporting self-congruity theory, research studying endorsers’ effect on promoting travel destinations found that having an image-congruent endorser enhances consumer attitude towards the ad (Xu et al., 2018). Similarly, research examining the similarity-attraction effect found that consumers prefer service providers similar to themselves in appearance features (Arndt et al., 2016). In addition, the influence of endorsers extends beyond shaping attitudes to affecting behavioral intentions. Surveys in the U.K. indicate that marketing initiatives, such as celebrity endorsements, have contributed to unplanned gambling expenditures (Wardle et al., 2022). Exposure to sports betting advertisements, especially those featuring promotional offers like free bets or prize draws, leads to increased gambling activity Hing et al. (2014). Building on these findings, this study proposes the following:

- H1. Sports betting ads using image-congruent endorsers generate stronger behavioral intentions towards the sportsbook than those using image-incongruent endorsers.

### Responsible Gambling and Sports Betting Advertisement

While sports betting generates additional tax revenue for state and local governments to improve residents’ quality of life, it could produce more harm than benefits to society because it comes with the costs of increased gambling problems (Nikkinen & Marionneau, 2020). From a social viewpoint, promoting games of chance, such as casino games, is accused of intensifying problem gambling (Lopez-Gonzalez et al., 2017). More importantly, sports betting advertisements often employ attention-grabbing strategies that are particularly appealing to adolescents, making them more susceptible to the message and more likely to engage in gambling, especially among boys (Botella-Guijarro et al., 2020; Nyemcsok et al., 2018; Pitt et al., 2018). Advertising literature shows that having exemplary public figures in the advertisement generates positive social effects, such as athlete endorsers effectively promoting healthier lifestyles (Brace-Govan, 2013). The dual entertainment path model proposes that celebrity endorsers shape consumer attitudes by triggering aspirational motives, encouraging consumers to emulate the endorsers’ characteristics or lifestyle (Hung, 2014; Karasiewicz & Kowalczuk, 2015). Aspirational motives arise from the alignment between an endorser’s image and a consumer’s ideal self—the version of themselves they aspire to become (Choi & Rifon, 2012). High-profile athletes, in particular, are often perceived as self-disciplined and morally upright, serving as influential role models for individuals with or aspiring to build a positive self-image (D. Kim et al., 2021). Building on these insights, this study proposes that audiences may demonstrate a stronger intention to behave responsibly when they align their self-image with the professionalism and moral integrity of celebrity endorsers featured in sports betting advertisements.

- H2. Endorser image congruence increases consumer intentions to gamble responsibly.

In 2023, sportsbook operators in the U.S. committed to promoting responsible gambling in their marketing communications by adhering to a code of conduct that mandates the inclusion of responsible gambling messages in advertisements. However, a post-event analysis of a state-wide responsible gambling awareness campaign in the U.S. reveals that such initiatives have limited impact unless audiences actively engage with the responsible gambling (RG) message (Williams et al., 2012). McCullock et al. (2024) found from a cross-sectional study that repeated RG message exposures lead to inattentiveness and psychological reactance. Given that celebrity endorsers can capture attention and enhance audience attitudes toward advertisements, such inattentiveness and reactance may be attenuated in celebrity-endorsed ads. Consequently, this may amplify consumer intention to gamble responsibly by increasing awareness of the RG messages. Building on these insights, this study proposes that the presence of an RG message and an image-congruent endorser jointly enhances responsible gambling intentions by increasing awareness of the RG message.

- H3. The presence of a responsible gambling message in the advertisement strengthens the effect of endorser-image congruence on consumers’ intention to gamble responsibly through increased awareness of the message.

### Endorser’s Race and Image Congruence

The congruence between celebrities and consumers can be measured from various perspectives, including gender, age, race, personality, and/or behavioral style (Alhabash et al., 2021; Choi & Rifon, 2012; Mundel & Yang, 2022; Xu et al., 2018). Given that U.S. sportsbooks seek to appeal to a diverse customer base, they have signed actors and athletes from various racial backgrounds as brand ambassadors. Mundel and Yang (2022) found that consumers respond more positively to advertising featuring the same race endorsers, demonstrating more favorable evaluations of the ad and stronger purchase intentions for the endorsed products. This evidence suggests that an endorser’s race may affect consumer perceived image congruence, with same-race endorsers generating a greater impact than different-race endorsers.

While the self-congruity theory contends that consumers prefer brands that resonate with their self-concept (Xu et al., 2018), social identity theory establishes that an individual’s self-concept is shaped by the social beliefs in the group (Hogg, 2016). The theory of conformity to social norms argues that society members often seek mental shortcuts by doing what everyone else in the group is doing or believes is right (Cialdini & Goldstein, 2004). Social norms are informal rules and standards beyond the law but can guide or constrain certain behaviors within a group (Melnyk et al., 2022). Individuals comply with social norms in pursuit of social harmony and a sense of belonging, as they seek social acceptance and aim to avoid being excluded (Li et al., 2022; Melnyk et al., 2022). A meta-analysis with 177 studies from 22 countries across 40 years concluded that social norms significantly affect consumer behavior across various cultures, and the effect does not vary by whether the behavior is private or public, hedonic or utilitarian (Melnyk et al., 2022). Research studying adolescent sports gambling behavior found that social norms significantly predict sports betting intentions, and the magnitude of the effect is even greater for players who are at high levels of problem gambling severity (Wang et al., 2021).

**Fig. 1:**
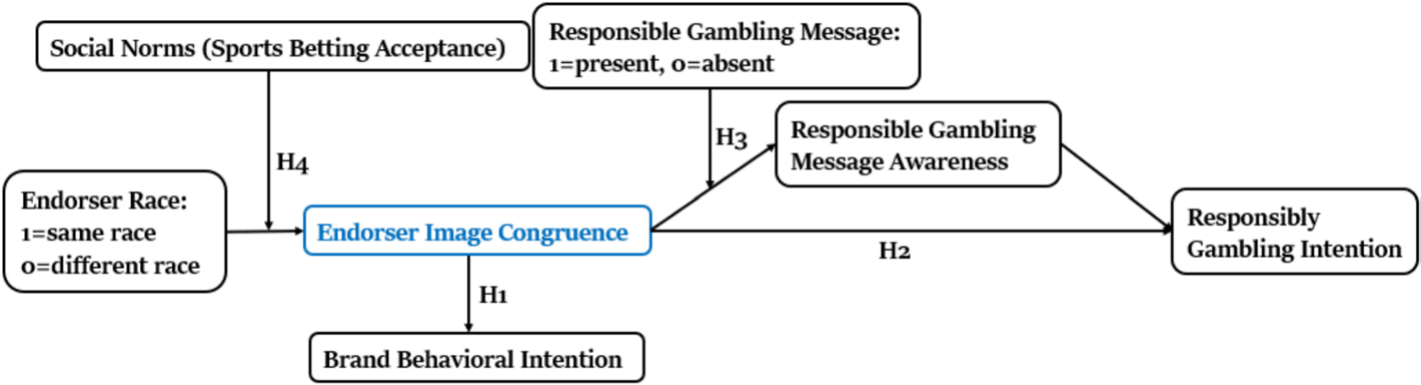
Conceptual Framework with Endorser Image Congruence as the Central Construct

Empirical evidence shows that advertising that does not comply with social norms or promotes socially disapproved behavior evokes negative emotions in consumers and leads to unfavorable brand evaluations (Noor et al., 2022). In addition, socially controversial ads featuring same-race endorsers are perceived as more negative and offensive than those with other-race endorsers, due to the perceived threat such portrayals pose to the group’s cultural identity (Terlutter et al., 2022). In general, celebrity endorsers who are perceived as highly congruent with the consumers are expected to enhance consumer attitudes and behavioral intentions toward the brand being promoted. However, this positive effect may reverse if the product being promoted is socially disapproved. Same-race endorsers may elicit negative reactions in such contexts, as consumers distance themselves to avoid associated stigma. While prior research has not addressed the impact of endorsers’ race on the effectiveness of sports betting advertisements, this study proposes that social norms moderate the relationship between endorsers’ race and perceived image congruence. Specifically, same-race endorsers are likely to be perceived as more congruent only when sports betting is socially accepted within the consumer’s social environment. When social acceptance is low, consumers may reject the association with same-race endorsers to maintain psychological distance and protect their identity. This study argues that endorser race and social norms interact to shape consumers’ perceptions of image congruence. Figure 1 presents the proposed conceptual framework and hypotheses.

- H4. Social norms moderate the effect of endorser race on consumers’ perceived image congruence. Specifically, compared to different-race endorsers, same-race endorsers will lead to higher perceived image congruence among consumers who perceive high gambling acceptance in their social environment, but lower perceived image congruence among those who perceive low gambling acceptance.

## Methodology

### Research Design and Respondents Recruitment

The study employed a 2 (endorser race: same race/different race × 2 (responsible gambling message: present versus absent) between-subjects scenario experiment design. Qualified respondents participated in an online survey where they were randomly assigned to review a sports betting ad with one of the four manipulation combinations and then asked to report their perceptions and behavioral intentions. The experiment stimuli were designed to mimic real-world practice. Therefore, the ads were adapted from real-life ads released by a well-known sportsbook in the U.S., with modifications using a fictitious brand name, LionBet. Two versions of the ads each featured a white or black celebrity athlete. As for responsible gambling messages, they can be in the form of advice, a slogan, or information on problem gambling prevention resources (McCullock et al., 2024). Following the Responsible Marketing Code for Sports Wagering (American Gaming Association, 2023b), the responsible gambling message stimuli in the current study contain the following information: Please gamble responsibly. Gambling problem? Call 1–800-xxx-xxxx.” The graphic stimuli used in the experiment can be found in the Appendix A.

The sample size of this experiment is determined by assuming a medium effect size of f = 0.25, an error probability of 0.05, and a power of 0.80. According to Cohen (1992), at least 215 participants are needed to reliably detect the interaction effects. Based on previous experience with data collection through online panels, a 45% attention check failure rate was assumed to ensure sufficient responses were acquired. Therefore, the experiment aimed to collect 400 responses from an online survey panel named Prolific.com. Prolific offers screeners, allowing researchers to filter participants by their demographics and interests. Two identical surveys were created to collect 200 responses each – one was restricted to White respondents, and the other was restricted to Black/African American respondents. The manipulation of the same race and different race endorsers was achieved by randomizing the two ads within these two surveys. Other selection criteria were the same for both surveys – participants were above 21 years of age and had self-reported engaging in online sports betting (See Appendix B for a screenshot of the filters used). Participants were recruited through Prolific and were told that they would be viewing ads for a sportsbook, the estimated duration of the study, and the monetary compensation for completing the survey. When interested participants clicked through to the survey, they were redirected to a Qualtrics survey, where they read an informed consent form as outlined in an IRB-approved protocol. Participants who provided their consent proceeded to the survey questions.

### Experiment Procedure

Participants first reported whether they had engaged in sports betting in the previous 12 months (Betting Experience: Yes or No), how often they participated in sports betting (Betting Frequency), and the largest amount they gambled in a single day (Largest Wager) (John, n.d.). This information was collected to ensure that the demographics on Prolific were accurate and current. Participants were then directed to view the ad for 5 seconds before measurement items appeared on the screen. (See Appendix C for the complete survey). They reported their attitude towards the ad (four items adapted from MacKenzie et al. (1986); Cronbach α = 0.96; one factor explaining 88.5% of the variance), and behavioral intention towards the brand (five items adapted from De Keyzer et al. (2022) and Singh and Banerjee (2018); Cronbach α = 0.93; one factor explaining 78.83% of the variance) on a 7-point Likert scales (1 strongly disagree… 7 strongly agree). An attention check was incorporated by instructing participants to select “somewhat agree” for a specific item. The ad remained on the screen as participants assessed endorser image congruence on a graphical scale adapted from Sen and Bhattacharya (2001), capturing their perceived congruence with the celebrity endorser in the ad. The respondents were then directed to the next screen where the ad was no longer presented and reported their awareness of the responsible gambling message (i.e., RG message awareness, Cronbach α = 0.93; one factor explaining 87% of the variance) and intention to gamble responsibly (i.e., RG intention, Cronbach α = 0.96; one factor explaining 93% of the variance) each on a three-item scale from Gilbert and Stafford (2024).

Based on the existing literature on factors affecting the effectiveness of celebrity endorsements (Yang, 2018), the survey included measurements of attitudes toward the endorser (Bergkvist et al., 2016), perceived expertise of the endorser on the product (Tzoumaka et al., 2016), and perceived endorser-brand fit (Xu et al., 2018). Respondents also responded to the 10-item problem gambling scale (i.e., South Oaks Gambling Screen) developed by Moore and Ohtsuka (1997) (Cronbach α = 0.95) and a three-item scale from Kape et al. (2024) capturing social norms around gambling (Cronbach α = 0.87; one factor explaining 79% of the variance). At the end of the survey, participants reported their demographic information, including age, gender, household income, highest level of education, and race. Participants were allowed to select multiple races first, and then were asked to choose only one that they considered themselves to be the most.^1^

## Results

### Sample

In total, 423 responses were collected. 23 participants failed the attention check. Five participants identified their race as other than White or Black/African American. 12 participants were flagged by Qualtrics as having potentially taken the survey multiple times. After removing the abovementioned 40 respondents, the final sample consisted of 383 participants with an average age of 37.67 (SD = 12.07). The sample comprised 185 Black/African American and 198 White; 125 women, 254 men, 2 non-binary, and 2 preferred not to state. Complete demographics are reported in Appendix D.

### Manipulation Checks

When the ad contained a responsible gambling (RG) message, respondents reported a greater level of awareness of the existence of the RG message (Mean_RG message present_ = 4.91, SD_RG message present_ = 1.41, N_RG message present_ = 182; Mean _RG message absent_ = 3.38, SD _RGmessage absent_ = 1.83, N_RG message absent_ = 201; *F* (1,381) = 82.59, *p* < 0.001).

### Main Analyses

#### Impact of Endorser Image Congruence

The effects of endorser image congruence on the dependent variables are presented in Table 1. Covariates refer to the control variables included in the regression model. The results of Behavioral Intention Model 1 support H1, showing that endorser image congruence has a significant positive effect on behavioral intentions toward the brand (*b* = 0.20, *se* = 0.05, *p* < 0.001). The results of Behavioral Intention Model 2 suggest that the presence of a responsible gambling message also has a significant impact on behavioral intentions (*b* = 0.55, *se* = 0.20, *p* < 0.01). However, the interaction effect is nonsignificant (*b* = −0.09, *se* = 0.07).

**Table 1:** Relationship between Dependent Variables and Endorser Image Congruence

Independent Variable	Behavioral Intention Model 1	Behavioral Intention Model 2	RG Intention Model 1	RG Message Awareness	RG Intention Model 2
Intercept	0.19 (0.59)	0.02 (0.59)	0.024 (0.74)	−0.85 (0.78)	−0.05 (0.80)
Image Congruence	0.15 (0.04) ***	0.20 (0.05)***	0.11 (0.05)^†^	0.39 (0.07)***	0.10 (0.07)
RG Message (1 = present, 0 = absent)	-	0.55 (0.20)**	-	2.34 (0.27)***	0.50 (0.28)^†^
Image Congruence x RG Message	-	−0.09 (0.07)	-	−0.29 (0.09)**	0.02 (0.09)
Covariates					
Age	0.00 (0.00)	0.00 (0.00)	−0.01 (0.01)	−0.01 (0.01)	−0.01 (0.01)
Gender	0.15 (0.11)	0.28 (0.10)**	−0.03 (0.14)	0.15 (0.14)	−0.04 (0.14)
Education level	0.06 (0.05)	−0.01 (0.04)	0.15 (0.06)**	0.16 (0.06)**	0.16 (0.06)
Household Income	0.04 (0.04)	−0.01 (0.04)	−0.04 (0.05)	−0.06 (0.05)	−0.04 (0.05)
Problem Gambling Index	0.12 (0.04)**	0.16 (0.04)***	0.38 (0.06)***	0.19 (0.05)***	0.38 (0.05)***
Betting Experience (1 = Yes, 0 = No)	−0.19 (0.22)	−0.47 (0.20)*	0.87 (0.28)**	0.27 (0.27)	0.81 (0.27)**
Betting Frequency	−0.03 (0.05)	−0.00 (0.05)	0.22 (0.06)***	0.00 (0.06)	0.21 (0.06)***
Largest Wager	−0.18 (0.07)**	−0.07 (0.07)	−0.48 (0.09)***	−0.1 (0.09)	−0.49 (0.09)***
Social Norm	0.07 (0.05)	0.20 (0.04)***	−0.02 (0.06)	0.04 (0.06)	−0.03 (0.06)
Attitude Towards the Celebrity	0.59 (0.07)***	0.47 (0.07)***	0.13 (0.09)	0.19 (0.1)*	0.11 (0.09)
Celebrity Expertise	0.16 (0.07)*	0.22 (0.06)***	0.22 (0.09)*	0.21 (0.08)*	0.20 (0.09)*
Celebrity Brand Fit	0.36 (0.07)***	0.20 (0.07)**	0.10 (0.09)	0.26 (0.09)**	0.13 (0.09)
Model Statistics					
Adjusted *R*^2^	0.53	0.541	0.295	0.475	0.321
*F* statistic	33.87	30.79	14.2	23.9	12.94
df1, df2	13, 366	15, 364	13, 366	15, 364	15, 364
*p-*value of the model	< 0.001	< 0.001	< 0.001	< 0.001	< 0.001

The entries in the table are the estimated regression coefficients with standard error in parentheses. ***p < 0.001, **p < 0.01, *p < 0.05, ^†^*p* < 0.1

Support for H2 is, at best, mixed. Endorser image congruence positively predicts intention to gamble responsibly (RG Intention), with a marginally significant effect (*b* = 0.11, *se* = 0.05, *p* = 0.052), as shown in the RG Intention Model 1 testing results. However, when the RG message is included as an additional predictor, neither the main effect of endorser image congruence nor its interaction with the RG message is significant (RG Intention Model 2 column of Table 1).

**Fig. 2:**
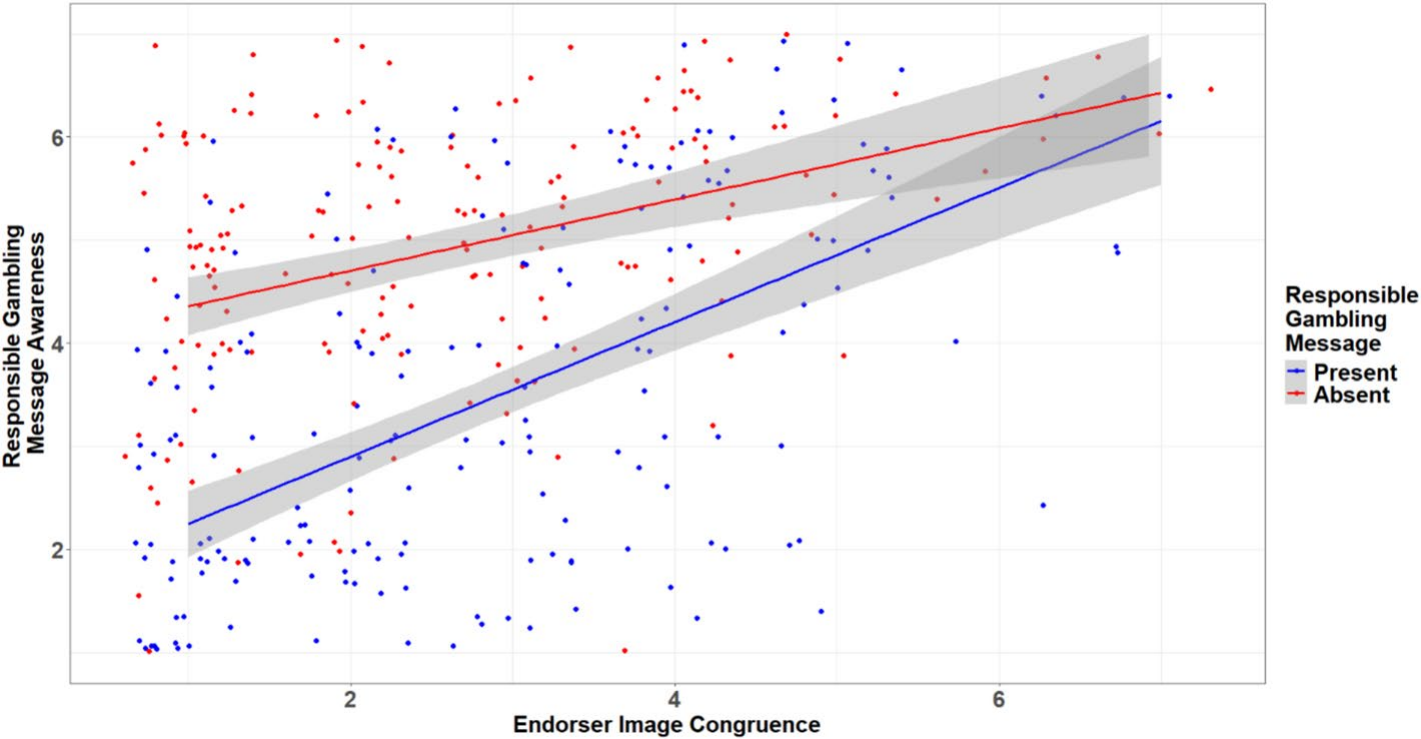
Conditional Effect of Image Congruence in Two RG Message Conditions

Surprisingly, the results indicate that endorser image congruence significantly increases RG message awareness (*b* = 0.39, *se* = 0.07, *p* < 0.001). Partially supporting H3, a significant interaction between the presence of an RG message and the perceived endorser-image congruence on RG message awareness is observed (*b* = −0.29, *se* = 0.09, *p* = 0.001). However, the direction of the interaction was contrary to expectations. While H3 proposed that the presence of an RG message would amplify the effect of endorser congruence, the negative interaction suggests otherwise. As illustrated in Fig. 2, individuals exposed to a highly congruent endorser reported high levels of RG message awareness regardless of whether the ad actually included an RG message. This implies that participants may have inferred the presence of an RG message simply due to the presence of a highly congruent celebrity endorser. These unexpected findings and their implications are further discussed in the conclusion.

**Fig. 3:**
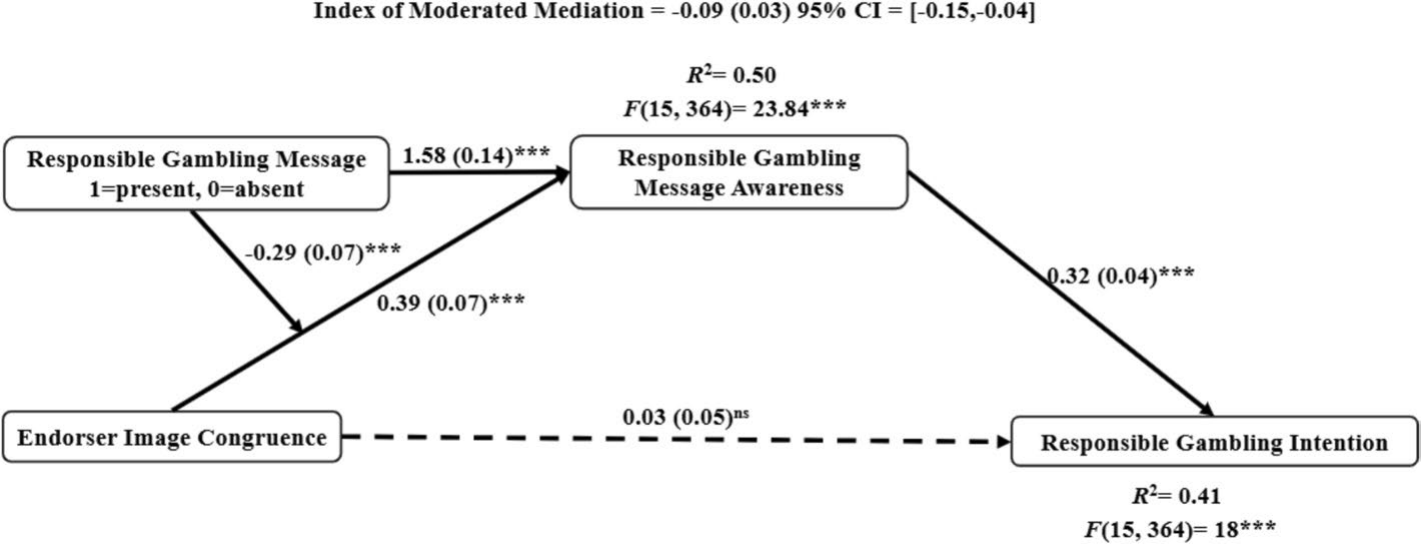
Endorser image congruence positively affects RG intention through RG message awareness. The presence of an RG message moderates the relationship between endorser image congruence and RG message awareness. Note: The dotted line represents a nonsignificant effect. The solid lines represent significant effects. ***p < 0.001

Next, a moderated mediation analysis examines the effect of endorser image congruence on RG intention, moderated by RG message presence and mediated through RG message awareness. The analysis uses PROCESS Macro Model 7 in SPSS, with 5,000 bootstrapped iterations and all control variables included. Figure 3 illustrates the model testing results. The direct effect of endorser congruence on RG intention is nonsignificant (*b* = 0.03, *se* = 0.05, *p* = 0.55). However, RG message awareness significantly predicts RG intention (*b* = 0.32, *se* = 0.04,* p* < 0.001). The main effect of endorser image congruence (*b* = 0.39, *se* = 0.07, *p* < 0.001), the presence of an RG message (*b* = 1.58, *se* = 0.14, *p* < 0.001), and their interaction term (*b* = −0.29, *se* = 0.07, *p* = 0.001) on RG message awareness are significant. As shown in Fig. 3, the positive relationship between endorser image congruence and RG message awareness is significant when RG message is absent (*b* = 0.39, *se* = 0.07, *p* < 0.001, 95% CI = [0.25,0.52]), but not when the RG message is present in the ad (*b* = 0.10, *se* = 0.07, *p* = 0.17, 95% CI = [−0.04,0.24]). The index of moderated mediation is significant (*b*** = **−0.09*, se* = 0.03, 95% CI = [−0.15,−0.04]) indicating that the indirect effect of endorser congruence on RG intention through RG message awareness is stronger when the RG message is absent (*b*** = **0.12*, se* = 0.03, 95% CI = [0.07,0.19]) and nonsignificant when the RG message is present (*b*** = **0.03*, se* = 0.02, 95% CI = [−0.003,0.07]). These findings suggest that endorser image congruence compensates for the absence of an explicit RG message in an advertisement. Full regression results appear in Table 2.

**Table 2:** Full Regression Results: Moderated Mediation Analysis Testing H3

Independent Variable	RG Message Awareness	RG Intention
Intercept	0.20 (0.79)	0.26 (0.77)
Image Congruence	0.39 (0.07)***	0.03 (0.05)
RG Message (1 = present, 0 = absent)	1.58 (0.14)***	–
Image Congruence x RG Message	−0.29 (0.09)**	–
RG Message Awareness	–	0.32 (0.04)***
Covariates		
Age	−0.01 (0.01)	−0.01 (0.01)
Gender	0.18 (0.14)	−0.07 (0.014)
Education	0.15 (0.06)**	0.09 (0.06)^†^
Household Income	−0.04 (0.05)	−0.02 (0.05)
Problem Gambling Index	0.19 (0.05)***	0.31 (0.05)***
Betting Experience (1 = Yes, 0 = No)	0.26 (0.27)	0.72 (0.26)**
Betting Frequency	−0.002 (0.06)	0.21 (0.06)***
Largest Wager	−0.11 (0.09)	−0.45 (0.09)***
Social Norm	0.04 (0.06)	−0.04 (0.06)
Attitude Toward the Celebrity	0.18 (0.09)*	0.05 (0.09)
Celebrity Expertise	0.22 (0.08)*	0.14 (0.08)^†^
Celebrity Brand Fit	0.25 (0.09)**	0.05 (0.08)

*N* = 380. The entries in the table are the estimated regression coefficients with standard error in parentheses. ***p < 0.001, **p < 0.01, *p < 0.05, ^†^*p* < 0.1. Model statistics for the RG Message Awareness *R*^2^ = 0.49, *F*(15, 364) = 23.84, *p* < 0.001. Model statistics for RG Intention *R*^2^ = 0.41, *F*(14, 365) = 18.0, *p* < 0.001

### Determinant of Endorser Image Congruence

**Fig. 4:**
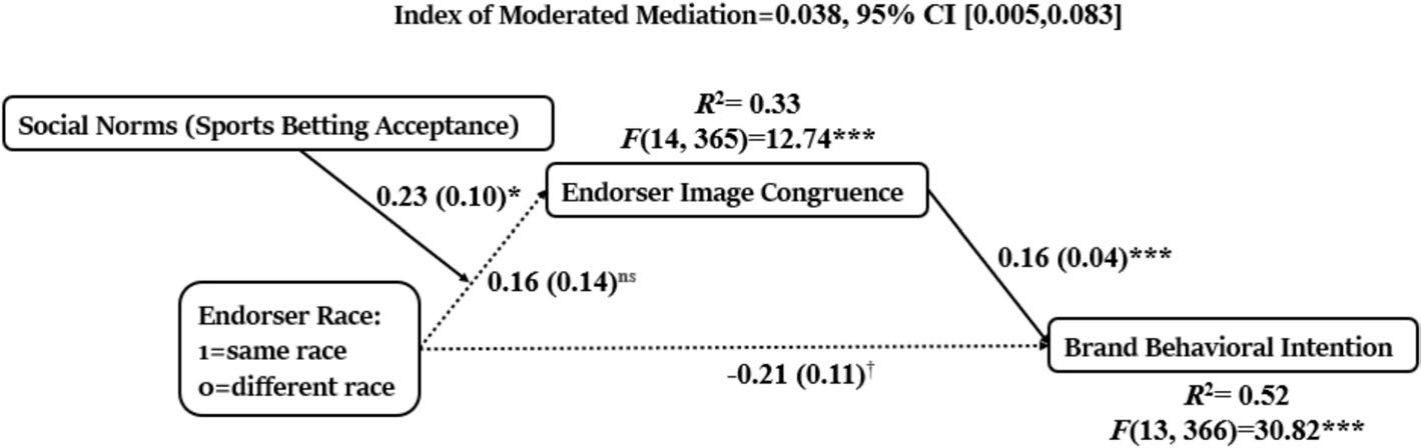
Endorser race affects brand behavioral intention through endorser image congruence when social norms suggest acceptance of sports betting. Note: The dotted line represents a nonsignificant/marginally significant effect. The solid lines represent significant effects. ***p < 0.001, *p < 0.05, ^†^*p* < 0.1

**Fig. 5:**
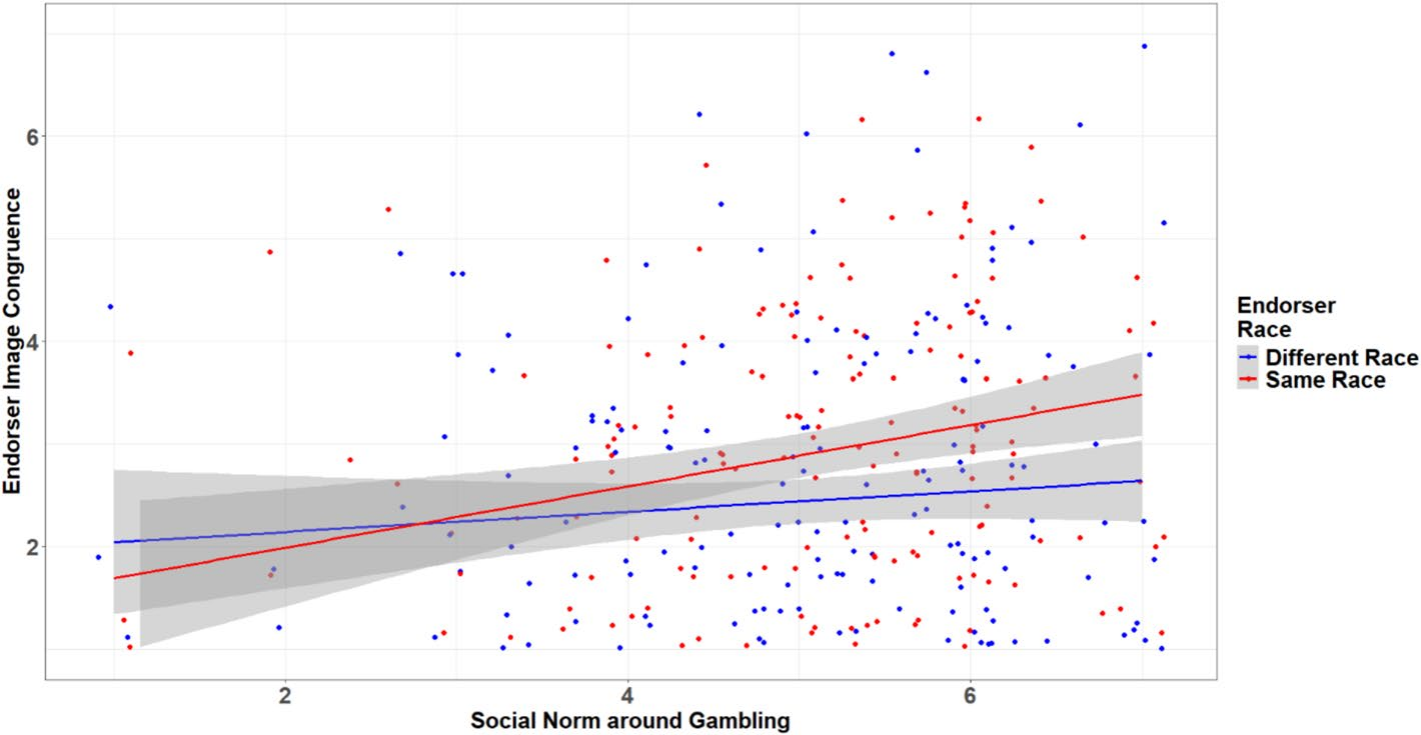
Conditional Effect of Social Norms in Two Endorser Race Conditions

The determinants of endorser image congruence are examined next. In the same-race endorser condition, respondents reported a higher level of perceived image congruence (Mean_same race_ = 2.86, SD_same race_ = 1.53, N_same race_ = 185; Mean_different race_ = 2.45, SD_different race_ = 1.55, N_different race_ = 198; *F* (1,381) = 6.80, *p* < 0.01). However, the magnitude of this difference is relatively small. H4 suggests that endorser race may serve as a lever to influence perceived image congruence, such that same-race endorsers are perceived as more congruent in contexts where gambling is socially accepted. The results in Table 3 support this postulate. While the main effects of endorser race (*b* = 0.16, *se* = 0.14, *p* = 0.22) and social norms (*b* = −0.06, *se* = 0.08, *p* = 0.47) on endorser image congruence are not statistically significant, their interaction is significant (*b* = 0.23, *se* = 0.10, *p* = 0.02), suggesting a moderation effect. A moderated mediation analysis is also conducted to examine whether endorser race indirectly influences behavioral intentions toward the brand through image congruence, with the effect moderated by social norms (Table 3, Fig. 4, 5). The direct effects of endorser race on image congruence (*b* = 0.16, *p* = 0.25) and brand behavioral intention (*b* =—0.21, *p* = 0.06) are nonsignificant. However, the path from endorser image congruence to brand behavioral intention is significant, *b* = 0.16, *p* < 0.001. The index of moderated mediation is significant, *b* = 0.038, 95% CI [0.005, 0.083], indicating that the indirect effect of endorser race on brand behavioral intention through image congruence varies as a function of social norms. Specifically, the conditional indirect effect is nonsignificant at low levels (Mean-1SD, *b* = −0.03, 95% CI [−0.09, 0.03]) and moderate levels (Mean, *b* = 0.03, 95% CI [−0.02, 0.08]) of social norms, but becomes significant at high levels (Mean + 1SD,* b* = 0.08, 95% CI [0.01, 0.17]) of social norms. Table 3 presents the full regression results.

**Table 3:** Full Regression Results: Moderated Mediation Analysis Testing H4

Independent Variables	Endorser Image Congruence	Brand Behavioral Intention
Intercept	−0.41 (0.71)	1.23 (0.56) *
Endorser Race (1 = Same, 0 = Different)	0.16 (0.14)	−0.21 (0.11) ^†^
Social Norms	−0.06 (0.08)	–-
Endorser Race x Social Norms	0.23 (0.10) *	–-
Endorse Image Congruence		0.16 (0.04) ***
Covariates		
Age	−0.01 (0.01)	−0.01 (0.005)
Gender	0.06 (0.14)	0.27 (0.11) *
Education	0.13 (0.06)*	−0.01 (0.04)
Household Income	0.09 (0.05) ***	−0.01 (0.04)
Problem Gambling Index	0.18 (0.05) ***	0.15 (0.04) ***
Betting Experience (1 = Yes, 0 = No)	−0.11 (0.26)	−0.69 (0.20) ***
Betting Frequency	−0.11 (0.06)	−0.01 (0.05)
Average Amount Wagered	−0.13 (0.09)	−0.03 (0.07)
Attitude Toward the Celebrity	0.44 (0.09) ***	0.52 (0.07) ***
Celebrity Expertise	0.17 (0.08) *	0.21(0.07) **
Celebrity Brand Fit	0.17 (0.09) *	0.25 (0.07) ***

N = 380. The entries in the table are the estimated regression coefficients with standard error in parentheses. ***p < 0.001, **p < 0.01, *p < 0.05, ^†^*p* < 0.1. Model statistics for Endorser Image Congruence R^2^ = 0.33, F(14, 365) = 12.74, p < 0.001, Model statistics for Brand Behavioral Intention R^2^ = 0.52, F(13, 366) = 30.82, p < 0.001

**Fig. 6:**
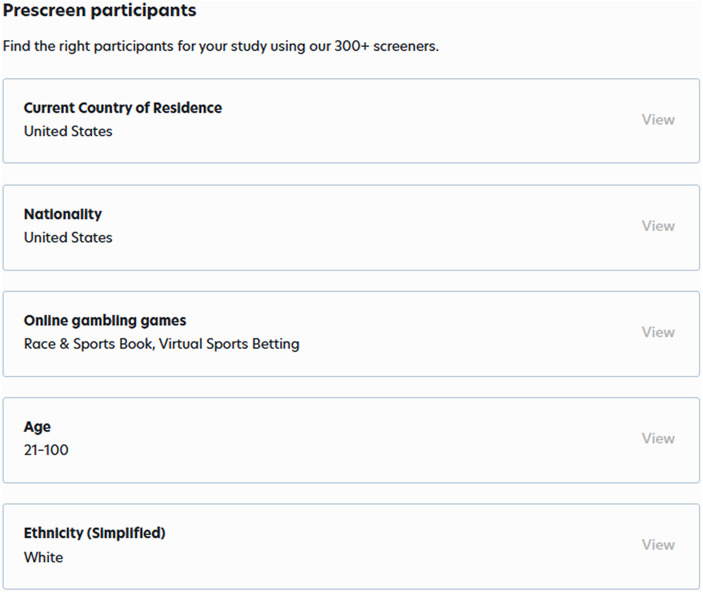
Screener used to recruit 200 participants who self-identified as White US nationals residing in the US, and had self-reported using race and sports books or engaging in virtual sports betting

For the path from endorser race to image congruence, there was a significant interaction between endorser race and social norms, *F*(1,365) = 5.39, *p* < 0.05, ΔR^2^ = 0.01. The positive conditional effect of endorser race on image congruence was significant at high levels of social norms (Mean + 1SD, *b* = 0.47, *p* < 0.05). It was nonsignificant at moderate level (Mean, *b* = 0.16, *p* = 0.25), and low levels of (Mean −1SD, *b* = −0.15, *p* = 0.43) of Social Norms. Figure 6 depicts the conditional effect of social norms at two conditions of endorser race, showing a significant positive effect of social norms when the same race endorser was present (*b* = 0.18, *p* < 0.05) and a nonsignificant effect when the different race endorser was present (*b* = −0.06, *p* = *0.45*).

## Discussion

### Conclusion

The purpose of the current study is threefold. First, it examines whether endorser–consumer image congruence enhances consumers’ behavioral intentions toward the sportsbook and their responsible gambling (RG) intentions in the context of sports betting. Second, the interaction between endorser image congruence and the presence of RG messages in sports betting advertisements is investigated to determine its effect on RG message awareness. Third, it examined whether the endorser’s race is a lever that impacts perceived image congruence and subsequent downstream behavioral intentions toward the sportsbook. It also examined the moderating role of social norms in the relationship between endorser race and image congruence. The data analyses revealed moderated mediation effects in both areas: a) the relationship between endorser image congruence and intentions to gamble responsibly, and b) the relationship between endorser race and brand intentions. Supporting H1, an image-congruent endorser enhances brand behavioral intentions, including the intention to learn more about the sportsbook brand, betting intentions, and word-of-mouth intentions. H2 is rejected, as an image-congruent endorser is found to have no significant direct effect on RG intentions. Contrary to expectations, H3 is only partially supported. Although the mediating effect of increased RG message awareness is confirmed, the hypothesized positive moderation effect of RG message presence on the relationship between image congruence and RG intention is not supported. Instead, the results reveal an opposite pattern: when endorser image congruence is high, the effect of RG messages on awareness is diminished, indicating a negative moderation effect. This should be interpreted alongside the unhypothesized finding that higher image congruence increases RG message awareness regardless of whether an explicit RG message is present in the ad. This suggests that image-congruent endorsers alone can enhance consumer awareness of responsible gambling, thereby appearing to weaken the additional impact of an RG message when both elements are present together.

Supporting H4, the findings suggest that same-race endorsers can enhance image congruence and consequently increase consumers’ brand behavioral intentions. However, the effect of endorser race is contingent upon social norms. While same-race endorsers significantly increased image congruence when sports betting is perceived as socially approved, a nonsignificant effect was found for individuals who perceive sports betting as having moderate or low social approval. Interestingly, in low social norm conditions, perceived image congruence for same-race endorsers was lower than for different-race endorsers, suggesting that the use of same-race endorsers in socially disapproved contexts may be perceived as offensive. These findings contribute to academic literature and industry practices, providing important insights for future research and strategic decision-making.

### Theoretical Contribution

Advertising plays an important role in promoting sports betting and normalizing gambling behavior (Lopez-Gonzalez et al., 2017). When more than one sportsbook dominates the market, advertising reinforces the audience about one particular brand (Lopez-Gonzalez et al., 2017). The findings of this research support the strategy of using celebrity endorsers in sports betting ads since they connect audiences to a lifestyle and social identity (Killick & Griffiths, 2023). The findings regarding consumer behavioral intentions deepen the understanding of celebrity endorsement effects on controversial products. While prior research suggests that advertisements are more effective in increasing purchase intention when featuring same-race endorsers (Mundel & Yang, 2022), this effect is conditional in the context of sports betting ads. When consumers perceive sports betting as socially approved, they are drawn to brands that align with their self-concept and use celebrity endorsers to construct an ideal self-identity (Boon & Lomore, 2001). However, when social norms surrounding sports betting are low, consumers do not perceive same-race endorsers as enhancing their self-concept, because an ideal self-identity fails to form due to societal disapproval of the behavior being advertised. This phenomenon may be explained by cognitive dissonance theory, which suggests that individuals adjust their beliefs when faced with threats to their self-concept to maintain a positive self-perception (Lambert-Swain, 2024). However, future research should study this in greater detail. In the current study, when respondents perceive sports betting as socially disapproved, they tend not to view the celebrity endorser as congruent with their ideal self-image. Unlike Terlutter et al. (2022), who found a negative advertising effect when same-race endorsers engaged in socially disapproved behavior, the current study did not find evidence supporting a negative impact of same-race endorsers when social acceptance of sports betting is low. However, the findings align with Terlutter et al. (2022) regarding the moderating effect of cultural differences on consumer ad perceptions, as the positive effect of same-race endorsers observed in a high social norms environment does not extend to contexts with low or moderate social acceptance.

The unhypothesized finding that image-congruent endorsers directly enhance RG message awareness raises an interesting research question and future research direction: Why do ad viewers perceive an RG message when it is not actually present? This phenomenon may be explained by the fuzzy-trace theory, which suggests that consumers can form false memories by associating the cues in the advertising with the brand (LaTour et al., 2014). The findings of this study imply that a celebrity’s established public image may contribute to false memories of RG messages. If a celebrity is perceived as an advocate for responsible gambling, viewers may subconsciously associate their image with an RG message, even when no such message appears in the advertisement. Another possible explanation is the halo effect, through which qualities associated with the endorser’s public image, such as discipline, control, and responsibility, are transferred to the product or activity being advertised (Yang, 2018). The positive attributes of celebrity endorsers can legitimize gambling behavior (Pitt et al., 2024), leading viewers to infer that if the activity were irresponsible or harmful, such respected figures would not be associated with it, thereby implying an element of responsibility. Nevertheless, further investigations are needed to understand the underlying mechanisms. Future research could explore the effect of endorsers on false memories of RG messages by examining potential mediators, such as consumer perceptions of endorser image, brand associations, and cognitive processes. Researchers may also examine the meaning transfer process to identify the presence of a potential halo effect.

One of the goals of the current study is to identify levers that could enhance the effectiveness of RG messages, as recent research found that RG messages are rarely noticed in advertising due to viewers’ psychological reactance and inattentiveness (McCullock et al., 2024). The findings of this study suggest that a highly image-congruent endorser enhances RG message awareness even when such messages are not present in the ad, which implies that image-congruent endorsers may have reduced the consumers’ psychological reactance to the notion of responsible gambling. However, future research is needed to find supporting evidence on such mechanisms. Scholars can also explore other factors that may reduce consumers’ psychological reactance and inattentiveness to RG messages.

### Practical Implications

The rapid growth of the sports wagering industry, coupled with intense competition among the four major sportsbooks—MGM, Caesars, DraftKings, and FanDuel—has driven extensive advertising efforts, particularly around major events like the Super Bowl and March Madness (Green, 2024). In line with their commitment to reducing gambling harm, businesses and organizations are working to raise awareness of responsible gambling while simultaneously promoting their brands. For example, ESPN launched “The Talk” campaign in January 2025, featuring Elle Duncan and Gary Striewski, to educate consumers on the importance of responsible gaming (Manzo, 2025). The findings of the current study contribute to this conversation and suggest that marketers can use image-congruent celebrities as a tool to promote responsible gambling.

While sports betting ads should encourage consumers to gamble responsibly, they also serve the goal of enhancing brand image and consumer intentions toward the brand. Marketers should not assume that endorsers of the same race as the target audience will always lead to better promotional results. Although same-race endorsers may enhance consumer resonance for regular products, this effect does not necessarily apply to a controversial product like sports betting. Marketers should consider the social norms and regional culture around sports betting before launching an ad or marketing campaign. Appealing to a target audience with same-race endorsers in a region where sports betting does not have high social acceptance might mitigate the effectiveness of the endorser. Sportsbooks should be cautious when selecting product endorsers for advertising to avoid potential backlash from consumers who may perceive the use of same-race endorsers as offensive. Nevertheless, future research should consider conducting cross-cultural studies to examine whether consumers in other countries exhibit similar behavior patterns. In addition to endorsers, other elements of advertising messages can significantly influence consumers’ behavioral intentions. Future research is encouraged to examine the interaction effects between image-congruent endorsers and promotional messages, such as bonus bets or reward expectancy, to enhance the effectiveness of sports betting advertising.

## Figures and Tables

**Fig. 7:**
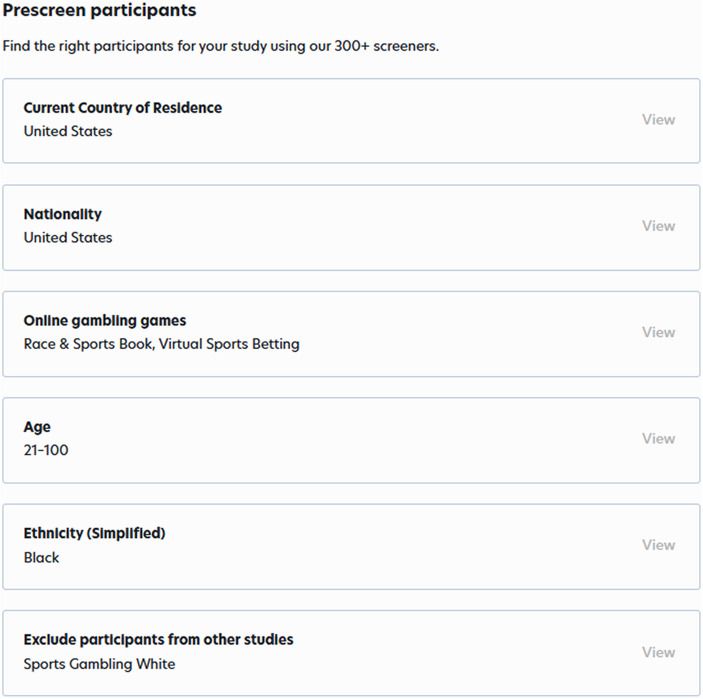
Screener used to recruit 200 participants who self-identified as Black US nationals residing in the US, and had self-reported using race and sports books or engaging in virtual sports betting. We ensured that participants who participated in the other study were excluded (to prevent bi-racial participants from potentially taking both studies)

## Data Availability

Data is available upon reasonable request.

## Appendix B: Filters used in Prolific

Two separate studies with distinct demographics filters were created using Prolific’s built-in screeners to target participants who had self-reported sports betting experience – one study targeted White participants, while the other targeted Black/African American participants. Figures 6 and 7 below are screenshots of the exact screeners used to select participants.

## Appendix C: Measurement Items

1. In the past 12 months, have you bet money on sports betting?^2^
  - Yes—344
  - No—39
2. How often do you participate in sports betting?
  - About every day—30
  - Several times a week—102
  - Once or twice a month—114
  - A few days a year—85
  - One day or less in the past 12 months—27
  - Less frequent than once several years—25
  - Several times a week—102
3. What is the largest amount of money you have ever gambled with any one day?

## ************************Respondents expose to Manipulations*********************

**Measurement Scale:** 7-point Likert Type Scale 1 = Strongly Disagree 2 = Disagree 3 = Somewhat Disagree 4 = Neither Agree nor Disagree 5 = Somewhat Agree 6 = Agree 7 = Strongly Agree.

## Advertising Attitude

1. I like this ad
2. This ad is appealing
3. This ad is interesting
4. This is a good ad

## Brand Intention^3^

1. I’m likely to place a bet using LionBet.
2. I would like to have more information on LionBet.
3. I’m interested in the LionBet brand.

## WOM Intention

1. I am likely to say positive things about LionBET to other people.
2. I am likely to encourage friends and relatives to bet through LionBET

## Endorser Image Congruence

Imagine that one of the circles (the one on the left) in each row represents your self-definition or identity, and the other circle (the one on the right in each row) represents the celebrity’s identity. Please indicate which row best describes the level of overlap between your own and the celebrity’s identity.

## Responsible Gambling Message Awareness

1. The ad reminds me to gamble responsibly.
2. The ad highlights tools and resources to help me gamble responsibly.
3. The ad teaches me about responsible gambling.

## Responsible Gambling Intention

1. After seeing the ad, I intend to reduce my average bet size.
2. After seeing the ad, I intend to gamble less frequently.
3. After seeing the ad, I intend to lower my gambling budget.

## Attitude towards the endorser

Earlier, you saw an ad for LionBet featuring a celebrity. What do you think about the celebrity who appeared in this ad?

## Expertise of the endorser

How much knowledge do you think the celebrity has about the product shown in the ad?

## Endorser-brand fit

How well do you think the celebrity fits with the brand?

## Problem Gambling Scale

1. To some extent, I have a gambling problem
2. I have, at times, gambled more than I meant to
3. People sometimes comment on the extent of my gambling
4. People sometimes criticize the amount I gamble
5. At times, I feel guilty about my level of gambling
6. I would like to cut down my level of gambling, but it’s difficult
7. I often try to win back the money I lose in gambling
8. Sometimes, I try to keep the amount I gamble secret from family or friends
9. On occasions, I have borrowed money to gamble or pay gambling debts
10. On occasions, I have taken time off school or work in order to gamble

## Subjective norms

1. Most people who are important to me think it is okay for me to gamble on sports.
2. Most people who are important to me understand that I gamble on sports.
3. Most people who are important to me agree with me about sports gambling

## Appendix D: Demographics

The following demographic questions were elicited from the participants.

1. How old are you?
2. In which state do you currently reside?
3. Choose one or more races that you consider yourself to be
  - White or Caucasian
  - Black or African American
  - American Indian/Native American or Alaska Native
  - Asian
  - Native Hawaiian or Other Pacific Islander
  - Other
  - Prefer not to Say
4. If you had to choose one race that you most consider yourself to be, which one would it be?
  - White or Caucasian
  - Black or African American
  - American Indian/Native American or Alaska Native
  - Asian
  - Native Hawaiian or Other Pacific Islander
  - Other
  - Prefer not to Say
5. What is the highest level of education you have completed?
  - Some High School or Less
  - High School Diploma or GED
  - Some college, but no degree
  - Associate or technical degree
  - Bachelor’s Degree
  - Graduate or Professional Degree (MA, MS, MBA, PhD, JD, MD, DDS etc.)
  - Prefer not to say
6. How would you describe your gender?
  - Male
  - Female
  - Non-Binary/third gender
  - Prefer not to say
7. What was your total household income before taxes during the past 12 months?
  - Less than $25,000
  - $25,000-$49,999
  - $50,000-$74,999
  - $75,000-$99,999
  - $100,000-$149,999
  - $150,000 or more
  - Prefer not to say

Table 4 presents the demographic distribution of the 383 participants in the main analyses.

**Table 4:** Demographic Distributions

Demographic Variable	Descriptives
Age	Mean = 37.67 SD = 12.07
Race [Single]	185 Black or African American, 198 White or Caucasian
Gender	254 Male, 125 Female, 2 Non-binary, 2 Prefer not to say
Household Income during the past 12 months	Less than $250,000–23 $25,000—$49,999–66 $50,000-$74,999–70 $75,000—$99,999–75 $100,000-$149,999–106 $150,000 or higher – 39 Prefer not to say—4
Education Level	High School Diploma or GED – 49 Some College but no degree – 53 Associate’s or Technical Degree – 35 Bachelor’s Degree – 151 Graduate or Professional Degree – 94 Prefer not to say—1

## Appendix E: Effect of Manipulation on Key Measures

Table 5

**Table 5:** Means (and SDs) of key variables across levels of manipulated variables

Endorser Race Matching	RG Message Presence	N	Ad Attitudes	Endorser Image Congruence	Behavioral intention	RG Message Awareness	RG Intention
Same Race	–-	185	4.90 (1.45)	2.87 (1.53)	4.67 (1.41)	4.17 (1.77)	3.36 (1.65)
Different Race	–-	198	4.71 (1.51)	2.45 (1.55)	4.53 (1.50)	4.05 (1.85)	3.15 (1.60)
–-	RG Message Present	182	4.93 (1.42)	2.59 (1.53)	4.72 (1.41)	4.91 (1.41)	3.52 (1.61)
–-	RG Message Absent	201	4.68 (1.53)	2.71 (1.57)	4.49 (1.49)	3.38 (1.83)	3.01 (1.61)
Same Race	RG Message Present	87	5.09 (1.44)	2.82 (1.52)	4.81 (1.35)	5.10 (1.36)	3.65 (1.66)
Same Race	RG message Absent	98	4.72 (1.45)	2.91 (1.54)	4.55 (1.45)	3.34 (1.84)	3.10 (1.61)
Different Race	RG Message Present	95	4.79 (1.40)	2.38 (1.52)	4.63 (1.46)	4.74 (1.44)	3.4 (1.57)
Different Race	RG message Absent	103	4.64 (1.61)	2.52 (1.58)	4.43 (1.53)	3.42 (1.82)	2.92 (1.60)

Results from Table 6 suggest that endorser race did not show any significant main effects, nor did it interact with the presence of the responsible gambling (RG) message.

**Table 6:** Relationship between the key independent variables (endorser race and presence of RG) message and key dependent variables (ad attitudes, brand intentions, RG message awareness, and RG intentions)

Independent Variables	Ad Attitudes	Brand Intentions	RG Message Awareness	RG Intention
Intercept	0.27 (0.63)	-0.02 (0.60)	-0.61 (0.81)	-0.14 (0.80)
RG Message: 1 = Present 0 = Absent	0.31 (0.15) *	0.34 (0.15) *	1.39 (0.20) ***	0.54 (0.19) **
Endorser Race: 1 = Same Race, 0 = Different Race	-0.20 (0.15)	-0.10 (0.15)	-0.30 (0.20)	0.07 (0.19)
Endorser Race x RG Message	0.04 (0.22)	-0.10 (0.21)	0.37 (0.28)	-0.002 (0.28)
**Covariates**				
Household Income	0.05 (0.04)	0.006 (0.04)	-0.03 (0.05)	-0.03 (0.05)
Gender	0.15 (0.11)	0.28 (0.11) **	0.17 (0.15)	-0.03 (0.14)
Education level	0.07 (0.05)	0.01 (0.04)	0.20 (0.06) ***	0.17 (0.06) **
Age	-0.001 (0.004)	0.002 (0.005)	-0.01 (0.007)	-0.01 (0.01)
Problem Gambling	0.13 (0.04) **	0.19 (0.04) ***	0.25 (0.05) ***	0.39 (0.05) ***
Social Norm	0.07 (0.05)	0.21 (0.04) ***	0.05 (0.06)	-0.02 (0.06)
Engaged in Sports betting in the past 12 months?	-0.18 (0.22)	-0.47 (0.21) *	0.27 (0.28)	0.80 (0.28) **
Sports Betting Frequency	-0.04 (0.05)	-0.012 (0.05)	-0.02 (0.06)	0.20 (0.06) **
Largest Amount Bet in a Single Day	-0.19 (0.07) **	-0.09 (0.07)	-0.13 (0.09)	-0.50 (0.09) ***
Attitude Towards the Celebrity	0.63 (0.07) ***	0.56 (0.07) ***	0.32 (0.09) ***	0.16 (0.09) ^
Perceived Celebrity Expertise	0.17 (0.07) **	0.25 (0.06) ***	0.24 (0.09) **	0.22 (0.09) **
Celebrity- Brand Fit	0.38 (0.07) ***	0.23 (0.07) ***	0.031 (0.09) ***	0.15 (0.09) ^
**Model Statistics**				
Adjusted R-squared	0.499	0.523	0.431	0.314
F (15,364)	26.18 ***	28.65 ***	20.14 ***	12.54 ***

The entries in the table indicate the regression coefficient (standard error)

*p*-values are denoted as follows *** *p* < 0.001, ** *p* < 0.01, * *p* < 0.05, ^ *p* < 0.1

## Footnotes

1 Nine respondents reported multiple races in the first selection; all other respondents identified themselves as mono-racial.

## Declarations

### Human Animal Right

All procedures performed in the current study involving human participants were in accordance with ethical standards. The current research was approved by the Purdue University Institutional Review Board (IRB#: IRB-2024–1818).
